# Whole-genome analysis of recombinant inbred rice lines reveals a quantitative trait locus on chromosome 3 with genotype-by-environment interaction effects

**DOI:** 10.1093/g3journal/jkad082

**Published:** 2023-04-13

**Authors:** Toshiyuki Sakai, Tomoaki Fujioka, Toyokazu Uemura, Shinichi Saito, Ryohei Terauchi, Akira Abe

**Affiliations:** Crop Evolution Laboratory, Kyoto University, Muko, Kyoto 617-0001, Japan; Iwate Agricultural Research Center, Kitakami, Iwate 024-0003, Japan; Aomori Prefectural Industrial Technology Research Center Agricultural Research Institute, Kuroishi, Aomori 036-0522, Japan; Fukushima Agricultural Technology Centre, Koriyama, Fukushima 963-0531, Japan; Crop Evolution Laboratory, Kyoto University, Muko, Kyoto 617-0001, Japan; Department of Genomics and Breeding, Iwate Biotechnology Research Center, Kitakami, Iwate 024-0003, Japan; Department of Genomics and Breeding, Iwate Biotechnology Research Center, Kitakami, Iwate 024-0003, Japan

**Keywords:** GxE, GWAS, QTL, rice, Plant Genetics and Genomics

## Abstract

Elucidating genotype-by-environment interactions is fundamental for understanding the interplay between genetic and environmental factors that shape complex traits in crops. Genotype-by-environment interactions are of practical importance, as they determine the performance of cultivars grown in different environments, prompting the need for an efficient approach for evaluating genotype-by-environment interactions. Here, we describe a method for genotype-by-environment detection that involves comparing linear mixed models. This method successfully detected genotype-by-environment interactions in rice (*Oryza sativa*) recombinant inbred lines grown at 3 locations. We identified a quantitative trait locus (QTL) on chromosome 3 that was associated with heading date, grain number, and leaf length. The effect of this QTL on plant growth–related traits varied with environmental conditions, indicating the presence of genotype-by-environment interactions. Therefore, our method enables a powerful genotype-by-environment detection pipeline that should facilitate the production of high-yielding crops in a given environment.

## Introduction

Understanding the genetic potential of crops is important for achieving higher yields. Quantitative trait locus (QTL) mapping and genome-wide association studies (GWASs) are popular approaches for identifying genetic factors controlling complex traits, and they have been used to identify major genomic regions associated with agronomically important traits in crops (Huang *et al*. 2010; Yano *et al*. 2019; Alqudah *et al*. 2020; Li *et al*. 2020). However, crop phenotypes are affected not only by genes but also by their interactions (epistasis) (Soyk *et al*. 2017; Tayefeh *et al*. 2018; Liu and Yan 2019; Sakai *et al*. 2021). In addition, the effects of genetics on some traits vary with environmental conditions, a phenomenon known as genotype-by-environment (GxE) interaction (El-Soda *et al*. 2014; de Leon *et al*. 2016). Identifying GxE interactions is important to better understand the factors controlling phenotypic variation in crops, which could serve as a guide for the cultivation of a genotype suitable to a given environment.

Rice (*Oryza sativa*) is an important staple crop worldwide. Grain yield in rice is affected by GxE interactions (Blanche *et al*. 2009; Sharifi *et al*. 2017). Whereas most studies have focused on identifying the environmental conditions that produce consistently high yields for a given promising rice cultivar, only a few have sought to identify specific genes or QTLs that are affected by environmental factors (Manneh *et al*. 2007; Ghandilyan *et al*. 2009; Xu *et al*. 2014). To further understand the effects of GxE interactions on rice traits, geneticists need a comprehensive, systematic method for GxE detection that employs whole-genome sequence information.

Statistical models and methods have been developed to identify GxE interactions (Malosetti *et al*. 2013; El-Soda *et al*. 2014). Various principal component analysis (PCA)–based approaches have been used to infer the adaptability of a given genotype to a specific environment; these approaches include the additive main effects and multiplicative interaction (AMMI) model and the genotype main effects and GxE (GGE) model (Zobel *et al*. 1988; Yan *et al*. 2000). Mixed models have also been developed to interpret GxE interactions in a large number of genotypes (Smith *et al*. 2005). QTL-by-environment interaction (QxE) models are useful for dissecting GxE interactions for individual genetic components (Malosetti *et al*. 2004; Thomas 2010; El-Soda *et al*. 2014). Several reports have proposed that recombinant inbred lines (RILs) and their genotype information are suitable for mapping genes and QTLs and for identifying loci with GxE interactions (Botto and Coluccio 2007; Ghandilyan *et al*. 2009; Fournier-Level *et al*. 2013; Phuong *et al*. 2019). However, these studies identified GxE interaction effects by comparing QTL mapping results across multiple environmental conditions or by applying statistical models incorporating only a limited number of markers. Therefore, statistical approaches are needed to directly identify GxE interaction effects using whole-genome sequence information specific to a particular RIL population.

In this study, we developed a linear mixed model incorporating genetic effects as a random polygenic effect to identify QTLs with GxE interaction effects in RIL populations. We focused on rice, first developing RILs and obtaining the whole-genome sequence of each RIL. We planted identical sets of RILs in 3 different locations to identify genomic regions that show GxE effects. We then compared the results of GWAS across multiple environments and applied linear mixed models based on single nucleotide polymorphism (SNP) genotypes of the RIL populations. Using this method, we successfully identified a rice locus with significant GxE interaction effects on heading date, grain number, and leaf length.

## Materials and methods

### Plant materials and growth conditions

**Fig. 1.**
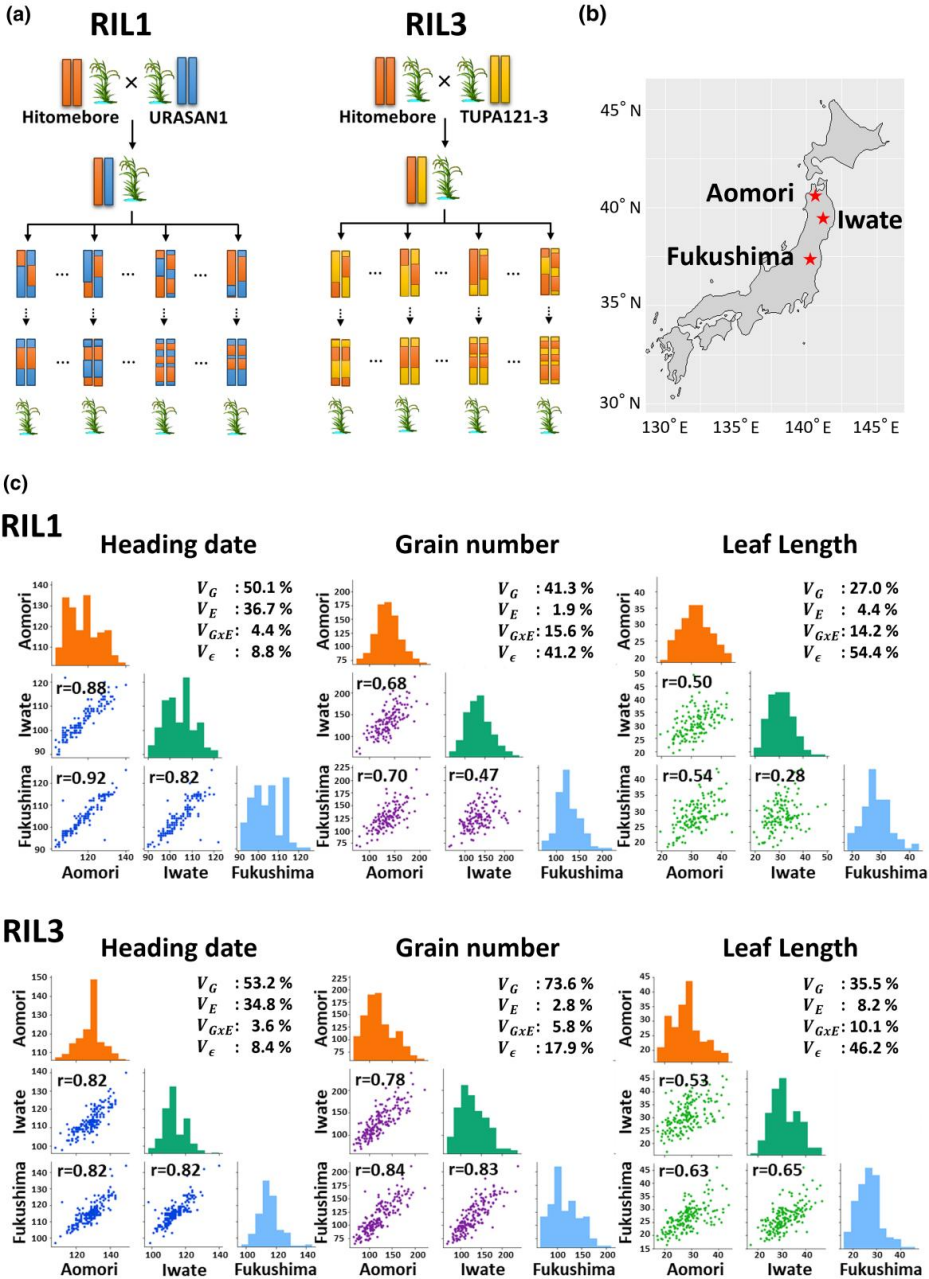
Correlation coefficients of phenotypic values among the trial locations. a) Scheme used to generate the RIL populations. b) Geographical map showing the locations of the trials: Aomori, Iwate, and Fukushima, Japan. c) Multiple scatterplots showing pairwise comparisons of the phenotypic values for heading date, grain number, and leaf length. Upper plots are for RIL1, and lower plots are for RIL3. Each scatterplot shows the relationships of phenotypic values between 2 of the 3 trial locations. The *y*-axis shows the phenotypic values of RILs at the first location, and the *x*-axis shows the corresponding values at the second location. Correlation coefficients (*r*) are shown above the plots. Diagonal histograms show the distribution of phenotypic values of RILs at each trial location. Each plot shows the proportion of variance components of genotype effect ($V_{G}$), environmental effect ($V_{E}$), GxE interaction effect ($V_{GxE}$), and residual effect ($V_{\varepsilon }$). These plots are based on the 2018 data set.

The *japonica* rice (*Oryza sativa*) cultivar “Hitomebore” was used as the common parent and was crossed to 5 cultivars: the *tropical japonica* rice cultivars “URASAN1” and “REXMONT”, the *aus* rice cultivars “TUPA 121-3” and “C8005”, and the *indica* rice cultivar “TAKANARI” from the National Agriculture and Food Research Organization World Rice Core Collection (Kojima *et al*. 2005) and our collection. For each cross, the resulting F1 plant was self-pollinated to obtain the F2 generation, which was used to generate the F9 generation by the single seed descent method (Fig. 1a). The code name and number of lines for each RIL are as follows: RIL1 (Hitomebore × URASAN1), 143 lines; RIL2 (Hitomebore × REXMONT), 187 lines; RIL3 (Hitomebore × TUPA121-3), 204 lines; RIL4 (Hitomebore × C8005), 248 lines; and RIL5 (Hitomebore × TAKANARI), 139 lines. These RIL populations were planted in 3 trial locations in Japan: Kuroishi (latitude 40°66′ N; longitude 140°59′ E, altitude 31 m) in Aomori Prefecture, Kitakami (latitude 39°35′ N; longitude 141°11′ E, altitude 89 m) in Iwate Prefecture, and Koriyama (latitude 37°47′ N; longitude 140°37′ E, altitude 217 m) in Fukushima Prefecture (Fig. 1b). Research trials with all RILs were conducted in 2018 and 2019. RIL1 was also studied in 2020. Fifteen seeds per RIL and their parents were germinated in water and sown in 2 × 2-cm plug trays filled with nursing culture soil. At 25–30 days after germination, seedlings from each RIL and parental line were transplanted into a paddy field (1 seedling per hill) at a density of 22.2–24.1 hills per m^2^. Basal fertilizer was applied at a rate of 30 kg N, 30 kg P, and 30 kg K per ha.

### Phenotyping

To elucidate the effect of GxE interactions on rice yields, we used grain number and heading date as yield-related phenotypes and leaf length as a plant growth–related phenotype. We counted grain number in a panicle on the main culm for all RILs in 2018 and 2019. Grain number of RIL1 was also measured in 2020. We also recorded heading date and leaf length for all RILs in 2018 and 2019. Heading date was counted as days to heading after sowing. The day when 50% of the individuals in a RIL showed panicle emergence was defined as the heading date of that RIL. Grain number was counted from a panicle in the main culm. Leaf length was measured using the flag leaf of the main culm. For all phenotypic data, 6 individuals per RIL were examined; their mean values were used for all analyses.

### Genotyping

To obtain the genotypes of the RILs, we performed whole-genome resequencing of the parental lines using the Illumina HiSeq platform with 150-bp paired-end reads and all the RILs using the Illumina NextSeq500 platform with 75-bp paired-end reads. We filtered and trimmed the short-read sequences using PRINSEQ (Schmieder and Edwards 2011) and FaQCs (Lo and Chain 2014). The quality-checked short reads were aligned against the reference genome using Burrows-Wheeler Aligner (Li and Durbin 2009). We used the sequence of Os-Nipponbare-Reference-IRGSP-1.0 as the reference genome (Kawahara *et al*. 2013). After mapping, we sorted and prepared index files from BAM files using samtools (Li *et al*. 2009). These BAM files were subjected to variant calling with bcftools (Narasimhan *et al*. 2016). Finally, we imputed the data from missing variants based on the genotypes of the parental cultivars using LB-impute (Fragoso *et al*. 2016). We identified 447,328 SNPs between the 2 parents for RIL1; 554,100 SNPs for RIL2; 1,518,711 SNPs for RIL3; 1,410,025 SNPs for RIL4; and 1,417,949 SNPs for RIL5. For QTL analysis and GxE detection, we selected 1 SNP per 10-kb interval and used 25,781 SNPs for RIL1; 29,521 SNPs for RIL2; 34,520 SNPs for RIL3; 32,379 SNPs for RIL4; and 32,256 SNPs for RIL5. These genotyping data were deposited in Zenodo (10.5281/zenodo.7213803).

### Environmental factors

To evaluate the environmental factors that vary across the 3 locations, we examined their soil composition and weather data. We sampled soil from these locations in spring 2019 before fertilization and measured pH, electrical conductivity (EC), cation exchange capacity (CEC), and chemical composition of available plant nutrients (NH_4_, NO_3_, CaO, MgO, K_2_O, and available phosphorus P_2_O_5_) in the soil. We also obtained weather data including temperature, precipitation, and sunshine duration (hours without cloud cover) for 2018 to 2020 from the AMeDAS data set of the Japan Meteorological Agency (Supplementary Tables 1 and 2).

### GWAS

To identify SNPs in the genomic regions harboring the major QTLs, we used a GWAS approach based on Mixed Linear Model analysis (Yu *et al*. 2006). We used the R package “GWASpoly” (Rosyara *et al*. 2016) to identify genomic regions that showed a significant association with the phenotypic effect. GWAS was performed separately on the phenotypic data for each RIL population in each location and each year to identify genomic regions significantly associated with a given trait. We calculated the false discovery rate (FDR) by Benjamini–Hochberg procedure based on the *P* values obtained by “GWASpoly” (Benjamini and Hochberg 1995). We extracted all SNPs with FDR < 0.01 that were significantly associated with the trait.

### Estimation of variance components and broad-sense heritability

To estimate the variance components of a random genotype effect, a random location effect as an environmental effect, and a GxE random effect, we considered the following mixed model as previously described (Jarquín *et al*. 2017):


y=μ+Zu+We+Vx+ϵ

$$u∼N(0,K\sigma_{u}^{2}),e∼N(0,\sigma_{e}^{2}),x∼N(0,[ZKZ^{\prime }]\circ [WW^{\prime }]\sigma_{x}^{2}),\epsilon ∼N(0,\sigma_{\epsilon }^{2})$$

where y is the n × 3-vector of phenotypic values from n samples at each of the 3 locations; μ is the overall intercept term; u is a vector of the random polygenic effect; $K\sigma_{u}^{2}$ is the variance of the random polygenic effect, where K is the additive genetic relationship matrix; e is the vector of a random environmental effect; $\sigma_{e}^{2}$ is the variance of the random environmental effect; x is the vector of the random effect in GxE interactions; $\sigma_{x}^{2}$ is the variance of the GxE component; ϵ is the residual error and $\sigma_{\epsilon }$ is the residual error variance; and Z,W,andV are the incidence matrices of 1s and 0s relating y to u, e, and x, respectively.

To estimate broad-sense heritability ($H^{2}$) based on estimated variance components, we used the following equation as previously described (Holland *et al*. 2010):


$$H^{2}=\frac{\sigma_{u}^{2}}{\sigma_{u}^{2}+\sigma_{e}^{2}/n_{e}+\sigma_{x}^{2}/n_{e}+\sigma_{\epsilon }^{2}/n_{e}}$$

where $n_{e}$ is the number of trial locations. The other symbols are described above.

After the identification of candidate SNPs with GxE effect, to estimate variance components of the random genotype effect, environmental effect, and the SNP genotype × environment interaction (SxE) random effect with a fixed SNP effect, we considered the following mixed model:


y=μ+Xβ+Zu+We+Sγ+ϵ

$$u∼N(0,K\sigma_{u}^{2}),e∼N(0,\sigma_{e}^{2}),\gamma ∼N(0,\sigma_{\gamma }^{2}),\epsilon ∼N(0,\sigma_{\epsilon }^{2})$$

where β is the fixed effect of a SNP; γ is the vector of the random effect of the genotype × environment interaction effect; $\sigma_{\gamma }^{2}$ is the variance of the genotype × environment interaction effect; and XandS are the incidence matrices of 1s and 0s relating y to β and γ, respectively. The other symbols are described above.

The models described above were fitted to the data and variance components were estimated using the BGLR package (Pérez-Rodríguez and de los Campos 2014). The script was deposited in GitHub (https://github.com/slt666666/GxE_analysis).

### Detection of GxE

To detect genomic regions that interact with environmental factors, we used a model comparison-based approach. We generated linear mixed models incorporating 1 SNP at a time sampled from the entire genome, with polygenic effect and environmental effect as the random effects. Model 2 considered the deviation of the effect of a given SNP by the trial location from the overall SNP effect, whereas model 1 did not. Models 1 and 2 were compared using the likelihood-ratio test to assess goodness-of-fit. Specifically, we considered the following linear models:


Model1:y=μ+Xβ+Zu+We+ϵ

$$u∼N(0,\;K\sigma_{u}^{2}),\;e∼N(0,\;\sigma_{e}^{2}),\;\varepsilon ∼N(0,\;\sigma_{\epsilon }^{2})$$

$$\mathrm{Model}\;2:y=\mu +X(\beta +We_{1})+Zu+We_{0}+\epsilon$$

$$\left(\begin{array}{c} e_{0}\\ e_{1} \end{array}\right)∼\left(\left(\begin{array}{c} 0\\ 0 \end{array}\right),\left(\begin{array}{c} \sigma_{e_{0}}^{2}\\ \sigma_{e_{0},e_{1}} \end{array}\begin{array}{c} \sigma_{e_{1}}^{2} \end{array}\right)\right)$$

where y is the n × 3-vector of phenotypic values from n samples at each of the 3 locations; μ is the overall intercept term; $e_{0}$ is the vector of a random environmental effect and $\sigma_{e_{0}}^{2}$ is the variance of the random environmental effect; $e_{1}$ is the random deviation of the effect of a given SNP by environment and $\sigma_{e_{1}}^{2}$ is the variance of the random deviation; and $\sigma_{e_{1},e_{0}}$ is the covariance of $e_{0}$ and $e_{1}$. The other symbols are described above.

In the likelihood-ratio test, the test statistic is −2logΛ, where Λ is the likelihood-ratio comparing models 1 and 2. The distribution of the test statistic is asymptotically a chi-squared distribution with degrees of freedom calculated as the difference of the number of parameters between models 1 and 2. Whether adding the GxE effect in model 2 leads to a performance gain that cannot be obtained by chance can be assessed by evaluating the distribution of the test statistic under the null hypothesis that the data are drawn only from model 1. When the likelihood-ratio test between models 1 and 2 showed statistically significant *P* values, adding the GxE effect of the SNP to the model (model 2) resulted in a significantly improved fit over model 1. Thus, we considered that the phenotypic value was affected by the interaction between the genomic region containing the SNPs and environmental factors. We calculated FDR by the Benjamini–Hochberg procedure based on the *P* values of the likelihood-ratio test of goodness-of-fit of all SNPs (Benjamini and Hochberg 1995). We extracted SNPs with FDR < 0.01 as candidate SNPs that significantly interact with the environment.

To fit a mixed-effect model to the data and to calculate the FDRs of the likelihood-ratio test, we used our original R scripts based on the R packages “lme4qtl” and “rrBLUP” (Endelman 2011; Ziyatdinov *et al*. 2018). The scripts were deposited in GitHub (https://github.com/slt666666/GxE_analysis).

### Analysis of environmental factors

To evaluate the environmental factors involved in GxE interaction effects in detail, we used a linear mixed model. We tested the significance of the coefficient for the interaction between each environmental factor and the genotype of the locus that was identified by the initial GxE analysis. We considered the following linear mixed model for each environmental factor (soil component or weather condition):


$$\mathrm{Model}\;3:y=\mu +X\beta +{E\beta }_{E}+Zu+\epsilon$$

$$\mathrm{Model}\;4:y=\mu +X(\beta +{E\beta }_{i})+{E\beta }_{E}+Zu+\epsilon$$

where E is an n-vector of environmental factor values for n samples, $\beta_{E}$ is the fixed effect of an environmental factor, and $\beta_{i}$ is the fixed effect of an interaction between the genotype of the locus and the environmental factor. The other symbols are described above. To evaluate the significance of $\beta_{i}$, models 3 and 4 were compared using the likelihood-ratio test as described for the GxE detection above. Whether adding the interaction between the genotype of the locus and the environmental factor in model 4 leads to a performance gain that cannot be obtained by chance can be assessed by evaluating the distribution of the test statistic under the null hypothesis that the data are drawn only from model 3. We calculated *P* values of the likelihood-ratio test for each environmental factor for each trait and each year. We calculated FDR by the Benjamini–Hochberg procedure based on the *P* values of all environmental factors (Benjamini and Hochberg 1995). We used our own R scripts based on the R packages “lme4qtl” and “rrBLUP.” To apply this model, we calculated the mean values of each weather condition data point for 0–30 days, 15–45 days, and 30–60 days prior to the heading date for each sample, year, and location. The scripts were deposited in GitHub (https://github.com/slt666666/GxE_analysis).

### Identifying the candidate gene associated with phenotypes involved in GxE interactions

To identify the gene with variable effects on heading date, grain number, and leaf length, we surveyed genes located in the genomic region identified by the GxE analysis (FDR < 0.01). We extracted genes with Trait Ontology (TO) terms related to the phenotypes based on the RAP-DB annotation as candidate genes (Sakai *et al*. 2013; Jupp *et al*. 2015). We used the following TO terms: days to heading (TO:0000137), flowering time (TO:0002616), days to maturity (TO:0000469), and inflorescence development trait (TO:0000621) for the phenotype of heading date; grain number (TO:0002759), filled grain number (TO:0000447), grain yield (TO:0000396), and nitrogen sensitivity (TO:0000011) for the phenotype of grain number; and leaf length (TO:0000135), leaf development trait (TO:0000655), and leaf shape (TO:0000492) for the phenotype of leaf length.

## Results

### Genetic effects on grain number vary depending on the trial location

To investigate whether the effects of genotype on phenotypes varied across the trial locations, we calculated the correlation coefficients of different phenotypic values of each RIL in each pair of trial locations. We also estimated the broad-sense heritability and the variance components of the genetic effect, the effect of location as the environmental effect, and the GxE effect for each RIL population. The RIL1 population showed a strong correlation for heading date among the 3 locations (r=0.82–0.94) with most of the phenotypic variance for heading date explained by genetic and environmental effects (Fig. 1c, Supplementary Tables 3 and 4). This result indicates that variation of heading date in RIL1 is solely based on the genotypes of the RIL and the trial location (environment). Other RIL populations also showed high correlation coefficients for heading date among trial locations, with similar proportions of variance components (Supplementary Tables 3 and 4). By contrast, grain number in RIL1 showed a lower correlation coefficient between the Iwate and Fukushima locations (r=0.47–0.61) (Fig. 1c, Supplementary Table 3). Grain number in the RIL2 population also showed a lower correlation coefficient (r=0.58–0.67) between Iwate and Fukushima (Supplementary Table 3). Estimated variance components in RIL1 and RIL2 indicated that over 10% of the phenotypic variance for grain number was explained by the GxE effect, suggesting that the variation in grain number in RIL1 and RIL2 is based not only on the genotype but also on the GxE interaction (Fig. 1c, Supplementary Table 4). However, grain number in the RIL3, RIL4, and RIL5 populations showed higher correlation coefficients (r=0.73–0.85) (Fig. 1c, Supplementary Table 3). All RIL populations showed lower correlations for leaf length among the 3 trial locations (r=0.28–0.76) with higher residual variance than other variance components (Fig. 1c, Supplementary Tables 3 and 4). Therefore, the variation for leaf length in the RIL populations is based not only on genotypes and trial locations. In summary, heading date appears to be mainly controlled by genetic factors and the trial locations (environment) in all RIL populations, whereas grain number might be controlled by both genetic factors and GxE interaction in RIL1 and RIL2, and leaf length might be controlled by not only genetic factors and the trial location but also factors unexplained by the statistical model.

**Fig. 2.**
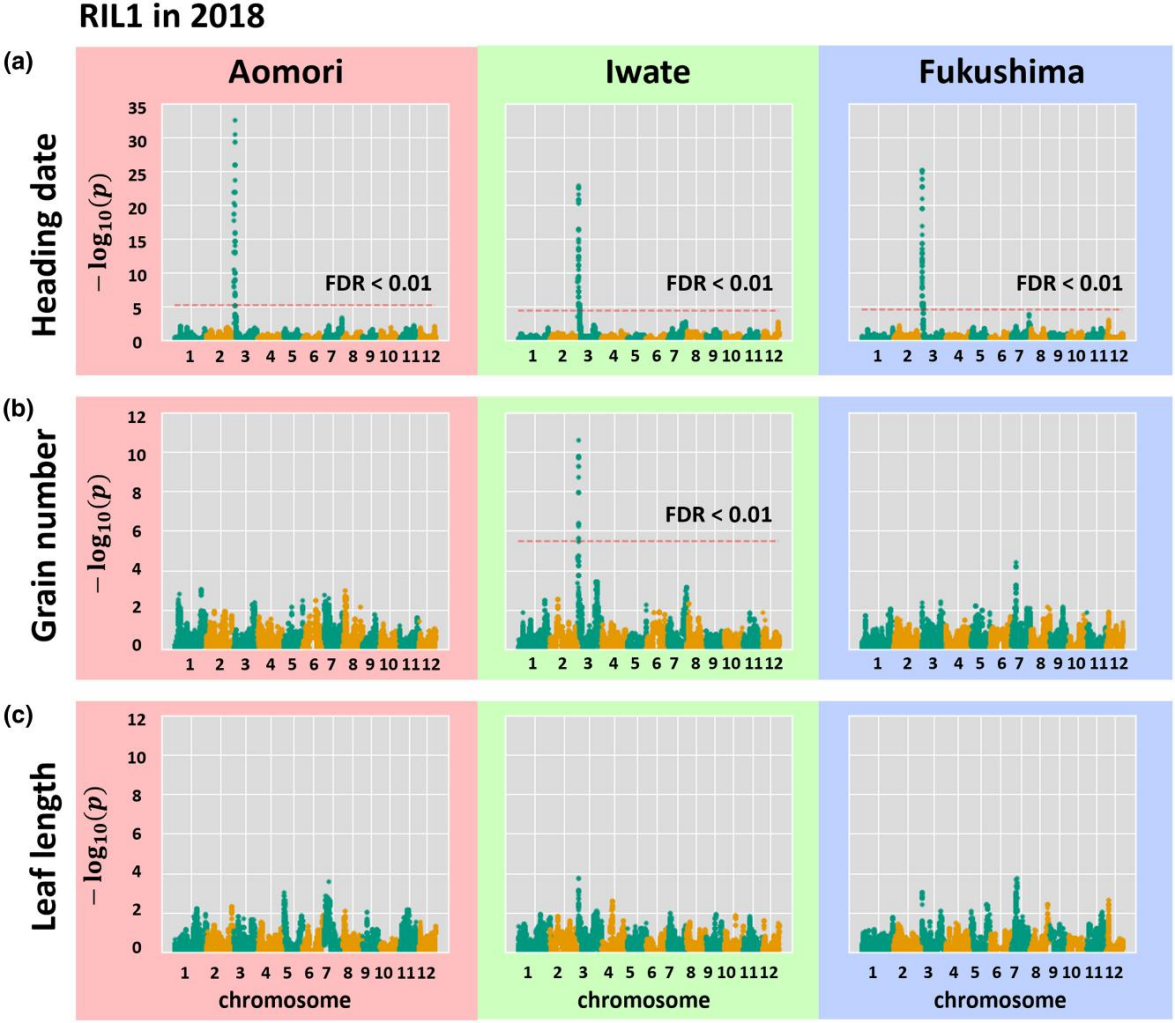
GWAS of heading date, grain number, and leaf length in the RIL1 population at the 3 trial locations. Manhattan plots showing the associations of SNPs with heading date a), grain number b), and leaf length c), as calculated by GWASpoly (Rosyara *et al*. 2016). The *y*-axis shows the −log10 (*p*) value of each SNP. The *x*-axis shows the genomic position. The dotted line indicates the significance threshold (FDR < 0.01). The results from each trial location are framed with different colors. Only SNPs located near chr03:1235072 exceeded the threshold FDR < 0.01 at all trial locations for heading date. SNPs located near chr03:1235072 exceeded the threshold only at Iwate for grain number. These plots are based on the 2018 data set.

To explore the genetic factors controlling the phenotypes of the RIL1 population at the 3 trial locations, we first conducted a GWAS separately for each location and attempted to identify the genomic regions involved in each phenotype. We identified a genomic region significantly associated with heading date (FDR < 0.01) on chromosome 3 (Fig. 2a, Supplementary Fig. 1, Table 5). This association was highly significant and was consistently identified at all 3 locations (Fig. 2a). By contrast, we detected no consistent genomic regions across the 3 locations associated with grain number or leaf length (Fig. 2, b and c, Supplementary Fig. 1, Table 5). However, at Iwate in 2018, we identified a genomic region on chromosome 3 significantly associated with grain number (Fig. 2b, Supplementary Table 5). The interval defined by this genomic region overlapped with that identified for heading date (Fig. 2, a and b). At Aomori in 2019, this genomic region was associated with leaf length (Supplementary Fig. 1, Supplementary Table 5). In summary, we identified a genomic region on chromosome 3 for heading date that also affected grain number and leaf length. However, we only detected this genomic region in 1 of the 3 locations for grain number. Therefore, the effect of the genomic region on grain number and leaf length appears to depend on environmental factors, suggesting the presence of a GxE interaction effect.

We also conducted a GWAS using other RIL populations. We identified genomic regions on chromosome 3 in RIL2 significantly (FDR < 0.01) associated with heading date at all 3 locations (Supplementary Fig. 1, Supplementary Table 5). This genomic region overlapped with that identified in the RIL1 population (Supplementary Fig. 1, Supplementary Table 5). These results indicate that heading date may be affected by the same genomic region in the RIL1 and RIL2 populations. We also identified genomic regions on chromosome 7 in RIL3 and chromosome 1 in RIL4 significantly (FDR < 0.01) associated with grain number at all 3 locations (Supplementary Fig. 1, Supplementary Table 5). Moreover, we identified genomic regions on chromosome 3 in RIL3 and on chromosome 7 in RIL5 for heading date and genomic regions on chromosomes 1 and 5 in RIL3 for leaf length. Each of these genomic regions was detected at a single location, although we observed weak peaks for *P* values over the same genomic regions at the other 2 locations (Supplementary Fig. 1). The results of this GWAS using the other RIL populations and other trial years are summarized in Supplementary Fig. 1 and Supplementary Table 5.

### A newly developed statistical method identifies a GxE interaction at a locus on rice chromosome 3

**Fig. 3.**
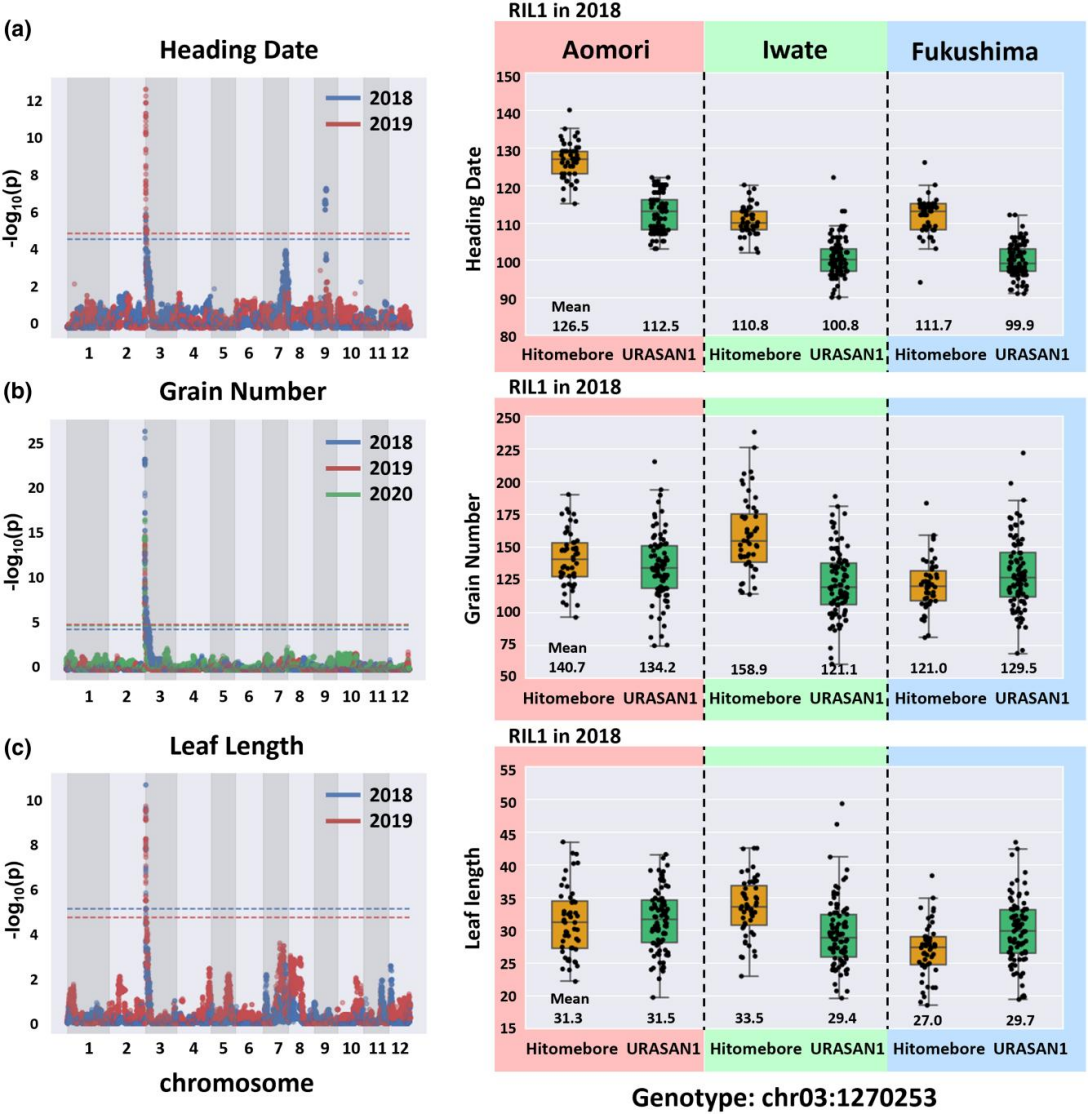
Identification of SNPs with GxE interaction effects and variation of genetic effects at the 3 trial locations. The Manhattan plots on the left show the positions of SNPs exhibiting significant GxE effects, as revealed by the likelihood-ratio test comparing 2 alternative models with/without a GxE interaction. The *y*-axis shows the −log10 (*p*) value of each SNP. The *x*-axis shows the genomic position. The colors indicate the trial years. The dotted lines indicate the significance threshold (FDR < 0.01). SNPs located near chr03:1235072 exceeded the threshold FDR < 0.01 for all traits. Boxplots on the right show the phenotypic values of RILs with different genotypes at SNP chr03:1235072 at the 3 trial locations. The horizontal line represents the median value. Box range is the first and third quartiles. The whiskers extend to the last data points less than the third quantile + 1.5 times the interquartile range (IQR) and the first data point greater than the first quantile −1.5 times the IQR. The *x*-axis shows the genotype of the SNP at chr03:1235072. The *y*-axis shows the phenotypic values. The results from each trial location are framed with different colors. Each row shows the result for each phenotype; a) Heading date. b) Grain number. c) Leaf length. These plots are based on the 2018 data set.

To detect GxE interactions affecting phenotypic traits, we applied a linear mixed model comparison-based approach to 25,781 SNPs in the RIL1 population distributed across the entire genome. After calculating *P* values for each SNP, we focused on those genomic regions with SNPs showing FDR < 0.01. We identified a genomic region on chromosome 3 (962,390–1,923,995 bp) as a candidate region showing significant GxE interaction effects for heading date in 2018 and 2019 (Fig. 3a, Supplementary Table 6). This genomic region overlapped with the QTL region identified by GWAS for heading date. We also applied this approach to grain number and leaf length and identified a genomic region on chromosome 3 (398,385–2,209,113 bp) as a candidate region showing significant GxE interaction effects for grain number in 2018, 2019, and 2020 (Fig. 3b, Supplementary Table 6). We also identified a genomic region on chromosome 3 (962,390–1,798,026 bp) as a candidate region showing significant GxE interaction effects for leaf length in 2018 and 2019 (Fig. 3c, Supplementary Table 6). These regions are close to the GxE region identified for heading date and the QTL for heading date (Fig. 2a).

To investigate the effects of the identified genomic region at the 3 locations, we plotted phenotypic values of the RIL1 population for the 2 possible genotypes at the SNP with the lowest *P* value, as revealed by the model comparison-based approach. We constructed a boxplot that shows the effect of the SNP at chr03:1235072 on heading date (Fig. 3a). When the genotype at this SNP was from the Hitomebore parent, the phenotypic values tended to be high at all 3 locations. In 2018, the effect of the SNP on heading date appeared to be greater at Aomori than at the other locations. By contrast, this locus did not have consistent effects on grain number or leaf length across the 3 locations (Fig. 3, b and c, Supplementary Fig. 2). In 2018, when the SNP at chr03:1235072 was from the Hitomebore parent, grain number tended to be high at Iwate but low at Fukushima (Fig. 3b). Grain number did not differ between the 2 possible genotypes for this SNP at Aomori (Fig. 3b). In 2019, when the SNP at chr03:1235072 was from the Hitomebore parent, grain number tended to be high at Aomori and Iwate and low at Fukushima (Supplementary Fig. 2). In 2020, when the SNP at chr03:1235072 was from the Hitomebore parent, grain number tended to be high at Aomori and Iwate, whereas we observed no great difference between the 2 possible SNPs at Fukushima (Supplementary Fig. 2).

Leaf length showed a tendency similar to that of grain number. In 2018, when the SNP at chr03:1235072 was from the Hitomebore parent, leaf length tended to be high at Iwate but low at Fukushima. Leaf length did not differ between the 2 possible SNPs at Aomori (Fig. 3c). In 2019, when the SNP at chr03:1235072 was from the Hitomebore parent, leaf length tended to be high at Aomori and Iwate (Supplementary Fig. 2). Leaf length did not differ between the 2 possible SNPs at Fukushima (Supplementary Fig. 2). These results indicate that the SNP at chr03:1235072 affects heading date, grain number, and leaf length and that the observed effects vary with location. Therefore, differences in environmental factors at the trial locations appear to be important for evaluating the genetic effect of the SNP at chr03:1235072 on heading date, grain number, and leaf length.

We also performed GxE analyses using the other RIL populations and identified 10 genomic regions showing significant GxE effects (Supplementary Fig. 3, Supplementary Table 6). Leaf length in RIL2 in 2018 showed a tendency similar to that in RIL1 across trial locations (Supplementary Fig. 4). Heading date of RIL2 in 2019 and RIL4 in 2018 also revealed a genomic region on chromosome 3 as a candidate region with a GxE effect. This genomic region was close to the region identified in RIL1 (Supplementary Figs. 3 and 4, Supplementary Table 6). Therefore, we observed a GxE effect associated with the genomic region on chromosome 3 identified in RIL1 for several traits in the other RILs. Heading date of RIL2 in 2019 also revealed other genomic regions on chromosomes 3, 7, and 10 as candidate regions with GxE effects (Supplementary Fig. 3). We also detected the genomic region on chromosome 10 for a GxE effect on heading date in RIL3 in 2019 (Supplementary Fig. 4). When the genotype at this genomic region was from the Hitomebore parent, the phenotypic values tended to be high at all 3 locations. The effect of this genomic region on heading date appeared to be greater at Aomori than at the other 2 locations for RIL2 and 3 (Supplementary Fig. 4). The effect of other candidate genomic regions on chromosomes 3 and 7 showed a similar tendency for heading date when the genomic region was from the Hitomebore parent: Heading date tended to be low at Aomori and Iwate with a greater effect than at Fukushima (Supplementary Fig. 4). Grain number of RIL4 in 2019 identified a genomic region on chromosome 1 as a candidate region with GxE effect (Supplementary Fig. 3). When the genotype at this SNP was from the C8005 parent, the phenotypic values tended to be high at all 3 locations. The effect of this genomic region on grain number appeared to be greater at Fukushima than at the other 2 locations (Supplementary Fig. 4). Leaf length in 2018 of RIL2 revealed a genomic region on chromosome 7 as another candidate region with GxE effect (Supplementary Fig. 3). When the genotype at this genomic region was from the REXMONT parent, the phenotypic values tended to be high only at Iwate (Supplementary Fig. 4). Heading date in 2018 of RIL5 revealed a genomic region on chromosome 9 as a candidate region with GxE effect (Supplementary Fig. 3). When the genotype at this genomic region was from the TAKANARI parent, the phenotypic values tended to be high at all 3 locations. The effect of this genomic region on heading date appeared to be greater at Fukushima than at the other 2 locations (Supplementary Fig. 4).

To assess the scale of the GxE effect identified by the above SNPs, we investigated the variance component of the identified SNP × environment interaction (SxE) effect in the phenotypic variance. We estimated the variance components of the genetic effect, the environmental effect, and the SxE effect for each candidate genomic region when considering a fixed SNP effect. The SxE effect of SNPs on chromosome 3 between 962,390 and 1,798,026 bp explained 3.2–4.8% of the phenotypic variance when excluding a fixed SNP effect for heading date (Supplementary Table 7). The SxE effect of this genomic region also explained 17.0–35.1% of the phenotypic variance excluding a fixed SNP effect for grain number and 22.8–31.2% for leaf length (Supplementary Table 7). These SNPs also showed similar variance components for the SxE effect on heading date and leaf length in RIL2 (Supplementary Table 7). The SxE effect of the SNP on chromosome 1 at 5,597,235 bp explained 7.6% of the phenotypic variance excluding a fixed SNP effect for grain number in RIL4 (Supplementary Table 7). The SxE effects of other candidate regions explained less than 3% of the phenotypic variance (Supplementary Table 7). In total, the estimated variance components suggested that the genomic region on chromosome 3 between 962,390 and 1,798,026 bp consistently shows a highly significant SxE effect on growth-related traits in RIL1 and RIL2. Therefore, we mainly focused on this genomic region for the investigation of candidate genes and important environmental components.

We surveyed genes located in the interval of chromosome 3 between 962,390 and 1,798,026 bp. This genomic region contains multiple genes that may be related to the phenotypes characterized in this study, such as *EARLY FLOWERING-COMPLETELY DOMINANT* (*Ef-cd*), *SUPPRESSOR OF OVEREXPRESSION OF CO 1* (*OsSOC1*), and *NIN-LIKE PROTEIN 1* (*OsNLP1*) (Supplementary Table 8). Genes that may be related to the phenotypes located in the other genomic regions identified by GxE analysis are summarized in Supplementary Table 8.

### Identifying environmental factors related to the GxE effect

Our GWAS and GxE analyses showed that the genotypic variation at the SNP on chromosome 3:1235072 affected heading date, grain number, and leaf length and that this genetic effect varied depending on the location (Fig. 3). To identify environmental factors with influence on the genetic effects of these 3 traits, we surveyed different environmental factors at the 3 trial locations. We obtained data for soil composition in the paddy field before fertilization in 2019 and weather condition data for 0–30, 15–45, and 30–60 days prior to heading date in the trial years for each location (Supplementary Tables 1 and 2). We generated a linear mixed model for each environmental factor to evaluate which environmental factors affect the genetic effect of the locus.

The lowest temperature during the 15–45 days prior to heading was a statistically significant environmental factor contributing to GxE effects of the chromosome 3 locus on heading date in RIL1, RIL2, and RIL4 (Supplementary Table 9). For both grain number and leaf length, sunshine duration showed a statistically significant effect during the 15–45 days and 30–60 days prior to heading (Supplementary Table 9). The highest temperature during the 30–60 days prior to heading was the most significant factor among weather conditions affecting heading date in 2019 (Supplementary Table 9). For grain number, precipitation during the 0–30 days prior to heading and temperature during the 15–45 days and 30–60 days prior to heading were statistically significant factors (Supplementary Table 9). Among soil properties, pH, EC, and NO_3_ and K_2_O concentrations were significantly associated with grain number and leaf length (Supplementary Table 9). Taken together, our GxE detection method revealed that the function of the locus on chromosome 3 is possibly affected by the lowest and highest temperatures, precipitation, sunshine duration, and the soil pH, EC, and NO_3_ and K_2_O concentrations at each location. The results of the analysis of environmental factors for other candidate genomic regions are summarized in Supplementary Table 9.

## Discussion

In this study, we identified a QTL on rice chromosome 3 with a GxE interaction effect for heading date and plant growth traits such as grain number and leaf length. We grew RIL populations at 3 locations and used GWAS as well as a linear mixed model comparison-based approach to identify a significantly associated genomic region with a genetic effect that varied with environmental factors.

This study demonstrates the power of our linear mixed model comparison-based approach to identify genomic regions involved in GxE interactions. Although our initial GWAS trial did not identify GxE interactions in heading date or leaf length, when we applied our new approach to these traits, we identified a locus with a GxE interaction effect for these 2 phenotypes (Figs. 2, a and c, 3, a and c). This result indicates that our approach has the power to detect a single locus with a significant GxE interaction effect when applied to well-genotyped RILs. Our approach focuses on 1 SNP at a time to address the effect of a GxE interaction. However, crop traits are usually controlled by multiple genes that interact with each other (Li *et al*. 1997; Wang *et al*. 2012; Sakai *et al*. 2021). Therefore, to predict GxE interactions involving multiple loci, a more complex model might be needed that incorporates additional variables including other QTLs and interaction effects (Malosetti *et al*. 2013).

We identified a QTL on chromosome 3 (1,235,072 bp) affecting heading date; this locus also showed a GxE interaction effect for heading date in the RIL1, RIL2, and RIL4 populations (Fig. 2a and 3, a and Supplementary Fig. 3). This genomic region contains the *Ef-cd* locus and *OsSOC1* (Supplementary Table 8). *Ef-cd* generates a long noncoding RNA that positively regulates the expression of the flowering activator gene *OsSOC1* (Lee *et al*. 2004; Bian *et al*. 2011; Fang *et al*. 2019). The *Ef-cd* allele, which was identified in *indica* rice line 6442S-7, accelerates maturation (Deng *et al*. 2002; Fang *et al*. 2019). Here, we showed that when the genotype of this locus was from the Hitomebore parent, heading date was delayed compared to the heading date conferred by the allele from the *tropical japonica* cultivar URASAN1 at all 3 trial locations (Fig. 3a). Our results are also in line with the finding of Fang *et al*. that the effect of *Ef-cd* on heading date depends on environmental factors (Fig. 3a) (Fang *et al*. 2019). Our results suggest that the *Ef-cd* locus of *tropical japonica* cultivar URASAN1 has a similar function as that of the *indica* cultivar.

The locus we identified also showed GxE interaction effects for grain number and leaf length (Fig. 3, b and c). Phenotypes related to plant growth and heading date are normally correlated because a long growth period leads to higher yields (Wang *et al*. 2018; Yu and Qian 2019). However, the *Ef-cd* locus causes a shortened maturation period with no yield penalty (Fang *et al*. 2019). We determined that when plants had the URASAN1 allele at this locus, their heading date was earlier compared to that of plants with the Hitomebore allele at all 3 trial locations (Fig. 3a). At Aomori and Fukushima, grain number and leaf length did not vary depending on heading date (Fig. 3, b and c), which is consistent with the finding that the *Ef-cd* locus accelerates maturation without a yield penalty (Fang *et al*. 2019). However, at the Iwate location, grain number and leaf length decreased with earlier heading date (Fig. 3). Therefore, our results suggest that the relationship between *Ef-cd*-mediated maturation period and plant growth traits is modulated by environmental factors. Alternatively, it is possible that other genes located in this genomic region have effects on plant growth traits, with GxE interaction effects.

Several genes that facilitate nitrogen utilization are upregulated via the function of the *Ef-cd* locus (Fang *et al*. 2019). Nitrogen utilization is closely related to the photosynthetic capacity and yield potential of crops (Chen *et al*. 2016, 2017; Wang *et al*. 2018). Our analysis showed that the soil NO_3_ concentration might contribute to the GxE interaction effect of our QTL for grain number and leaf length (Supplementary Table 9). It is possible that the concentration of available nitrogen in the soil influences the genetic effects of the *Ef-cd* locus on grain number and leaf length. However, the genomic region identified by our GxE analysis also contains *OsNLP1* (Supplementary Table 8), which enhances nitrogen utilization to improve plant growth and grain yield under nitrogen limitation conditions (Alfatih *et al*. 2020). Therefore, *OsNLP1* is also a candidate with a possible GxE interaction effect on grain number and leaf length in the RIL1 population.

Several genes of unknown function are also located in the genomic region we identified. Furthermore, our analysis of environmental factors revealed several factors that showed significant GxE interaction effects, including lowest and highest temperatures, precipitation, and sunshine duration (Supplementary Table 9). Therefore, additional experiments under more controlled environmental conditions are needed to identify gene sets and environmental factors that contribute to the GxE interaction. Identifying relevant environmental factors is easier under controlled laboratory conditions than under natural conditions; however, controlled conditions do not reflect the natural environments where crops are actually grown (Anderson *et al*. 2014; de Leon *et al*. 2016). Thus, to understand the genetic and environmental factors involved in GxE interactions, it will be important to measure the effects of environmental factors under both natural and controlled laboratory conditions (Xu 2016; Xu *et al*. 2017).

Our results show that the effects of QTLs on yield traits such as grain number vary with environmental factors (Fig. 3b). Numerous GWASs have identified QTLs associated with agronomically important traits in rice (Huang *et al*. 2010; Yano *et al*. 2019). However, most of these studies did not consider environmental effects or GxE interaction effects. Therefore, utilizing our approach to elucidate GxE interactions for the QTLs identified in these studies could potentially identify strategies to improve crop yields.

In summary, we used a linear mixed model comparison-based approach to identify a locus on rice chromosome 3 showing GxE interaction effects on heading date, grain number, and leaf length. This genomic region showed genetic effects on phenotypes related to plant growth and yield traits, and these effects varied with environmental factors. These results shed light on the genetic mechanism underlying the effects of GxE interactions on plant growth traits. In addition, our approach could facilitate the selection of appropriate combinations of rice cultivars for cultivation at different locations.

## Supplementary Material

jkad082_Supplementary_Data

## Data Availability

Genotype, phenotype, weather, and soil composition data were deposited in Zenodo (10.5281/zenodo.7213803). Scripts for GxE analysis were deposited in GitHub (https://github.com/slt666666/GxE_analysis). Supplemental material available at G3 online.
