# Supplementary material for: Whole-genome analysis of recombinant inbred rice lines reveals a quantitative trait locus on chromosome 3 with genotype-by-environment interaction effects
Source: G3 (Bethesda). 2023 Apr 13;13(6):jkad082. doi: 10.1093/g3journal/jkad082 (PMC10234396; doi:10.1093/g3journal/jkad082)
Supplement: jkad082_Supplementary_Data [file jkad082_supplementary_data.zip › Supplemental_Figures_G3-2023-404187.pdf]

Supplemental figures

Whole-genome analysis of recombinant inbred rice lines reveals a QTL on chromosome 3 with genotype-by-environment interaction effects

Toshiyuki Sakai[1*], Tomoaki Fujioka[2], Toyokazu Uemura[3], Shinichi Saito[4], Ryohei Terauchi[1], Akira Abe[5*]

[1] Crop Evolution Laboratory, Kyoto University, Muko, Kyoto, 617-0001, Japan
[2] Iwate Agricultural Research Center, Kitakami, Iwate 024-0003, Japan
[3] Aomori Prefectural Industrial Technology Research Center Agricultural Research Institute, Kuroishi, Aomori, 036-0522, Japan
[4] Fukushima Agricultural Technology Centre, Koriyama, Fukushima, 963-0531, Japan
[5] Iwate Biotechnology Research Center, Kitakami, Iwate 024-0003, Japan

*Corresponding Author
Toshiyuki Sakai, Email: sakai.toshiyuki.3w@kyoto-u.ac.jp
Akira Abe, Email: a-abe@ibrc.or.jp

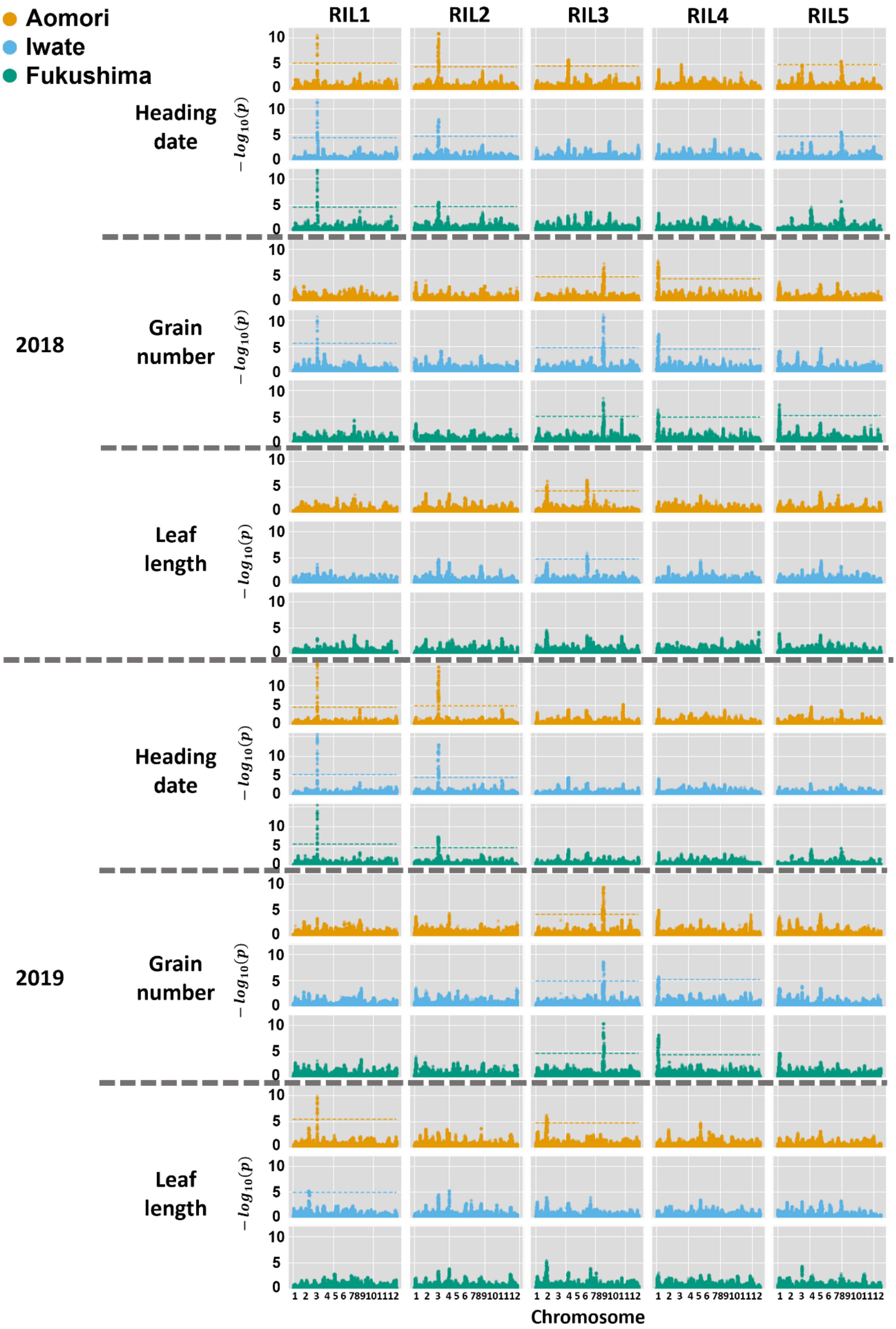

Aomori
Iwate
Fukushima
RIL1
RIL2
RIL3
RIL4
RIL5
2018
2019
Heading date
Grain number
Leaf length
$-log_{10}(p)$
10
5
0
1 2 3 4 5 6 789101112
Chromosome

Figure S1 Quantitative trait locus analysis of heading date, grain number, and leaf length for all trial years and all RIL populations. Manhattan plot showing the significant association of SNPs with phenotypes as calculated by GWASpoly (Rosyara et al. 2016). The y-axis shows the –log10 (p) value of each SNP. The x-axis shows the genomic position. The dashed line indicates the significance, i.e., FDR <0.01. Each row indicates each year, each phenotype, and each trial location. Each column indicates each RIL population. Each color of plot indicates each trial location.

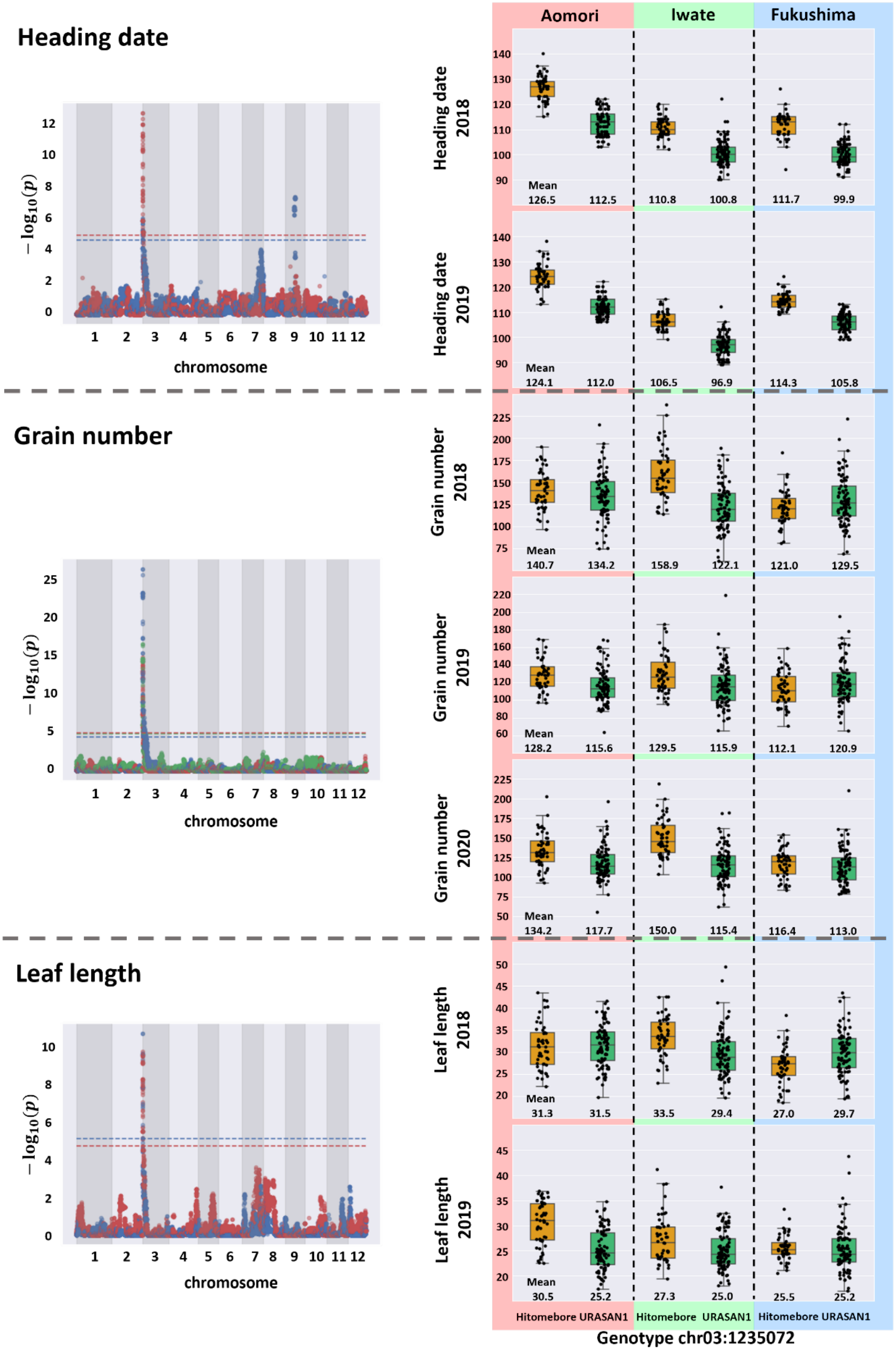

Figure S2 Identification of SNPs with GxE interaction effect in RIL1 and the variation of

genetic effects in three locations for all trial years. The left Manhattan plots shows the position of SNPs exhibiting GxE effect with statistical significance as revealed by the loglikelihood test comparing two alternative models with/without GxE interaction. The y-axis shows the –log10 (p) value of each SNP. The x-axis shows the genomic position. The colors show the trial years. The dashed lines indicate the significance, i.e., FDR <0.01. Boxplots in the right show the phenotypic values of RILs in three trial location with different SNP types at the genomic position identified in the left Manhattan plot. The horizontal line inside the box represents median value. Box range is the first and third quantile. The whisker extends to last datum less than the third quantile + 1.5*interquartile range (IQR) and the first datum greater than the first quantile—1.5*IQR. The X-axis shows the genotype of the SNP. The Y-axis shows phenotypic values. Each color frame indicates results from each trial location.

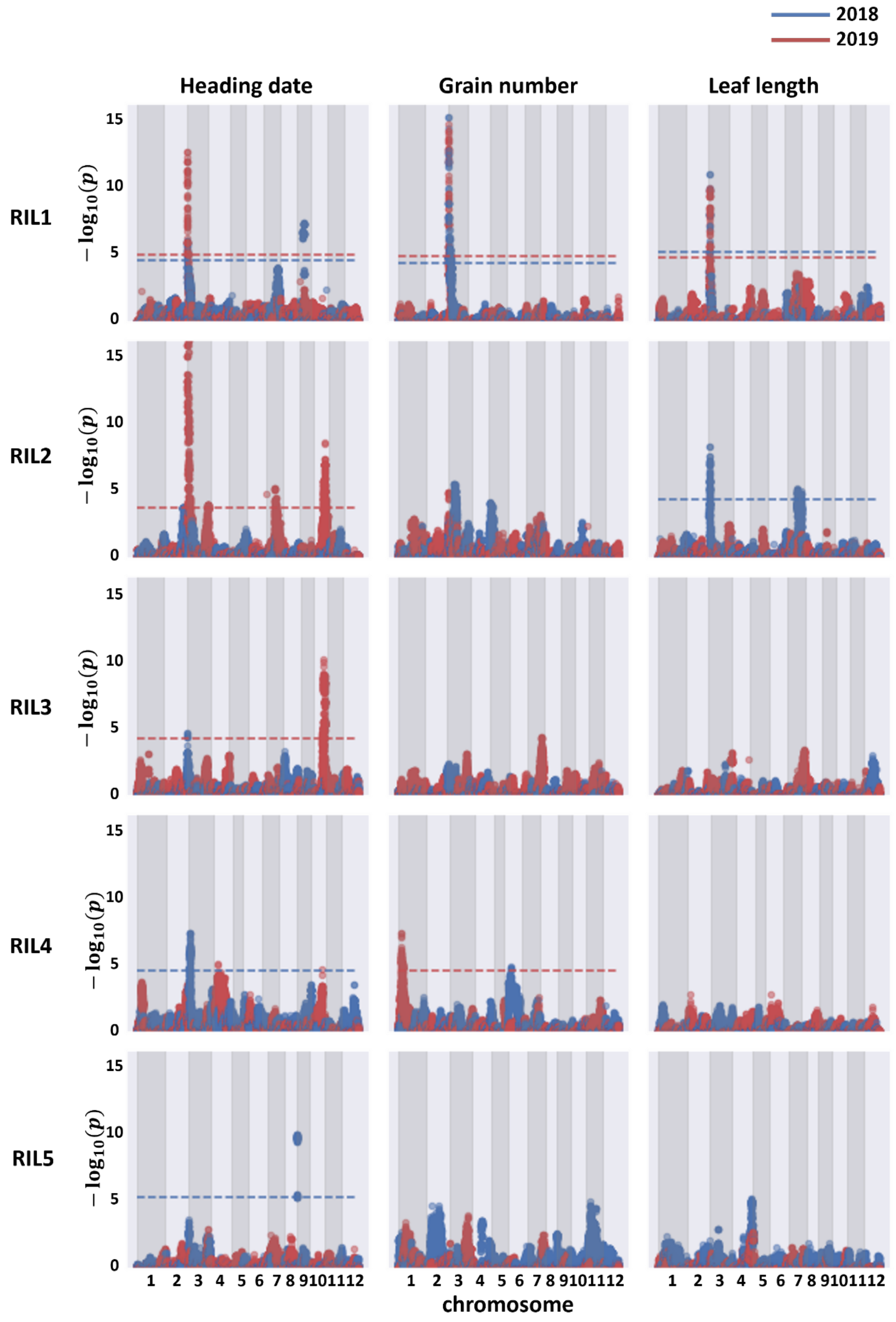

Figure S3 The identification of SNPs with GxE interaction effect. The Manhattan plots

showing SNPs with statistical significance in the loglikelihood test that compare models with/without GxE interaction effect. The y-axis shows the –log10 (p) value of each SNP. The x-axis shows the genomic position. Each color shows the trial year. The blue dashed line indicates the significance, i.e., FDR <0.01 in 2018 data. The red dashed line indicates the significance, i.e., FDR <0.01 in 2019 data. SNPs located near chr03:1235072 exceeded the threshold FDR <0.01 in 2018 for heading date in RIL2 and 2019 in RIL4, and in 2019 for leaf length phenotype in RIL2.

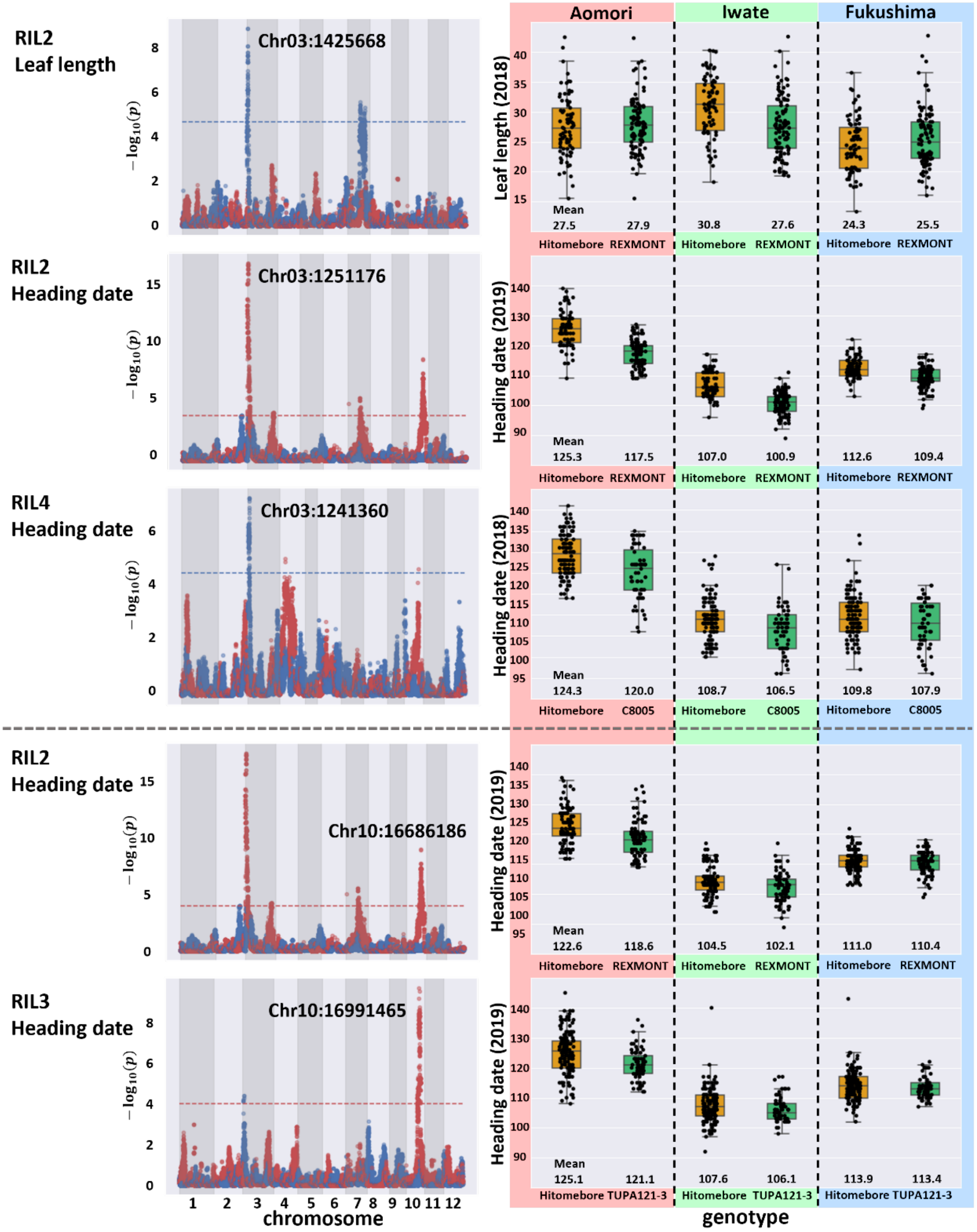

RIL2
Leaf length
Chr03:1425668
RIL2
Heading date
Chr03:1251176
RIL4
Heading date
Chr03:1241360
RIL2
Heading date
Chr10:16686186
RIL3
Heading date
Chr10:16991465
chromosome
Aomori
Iwate
Fukushima
Leaf length (2018)
Heading date (2019)
Heading date (2018)
Mean
Hitomebore
REXMONT
C8005
TUPA121-3
genotype

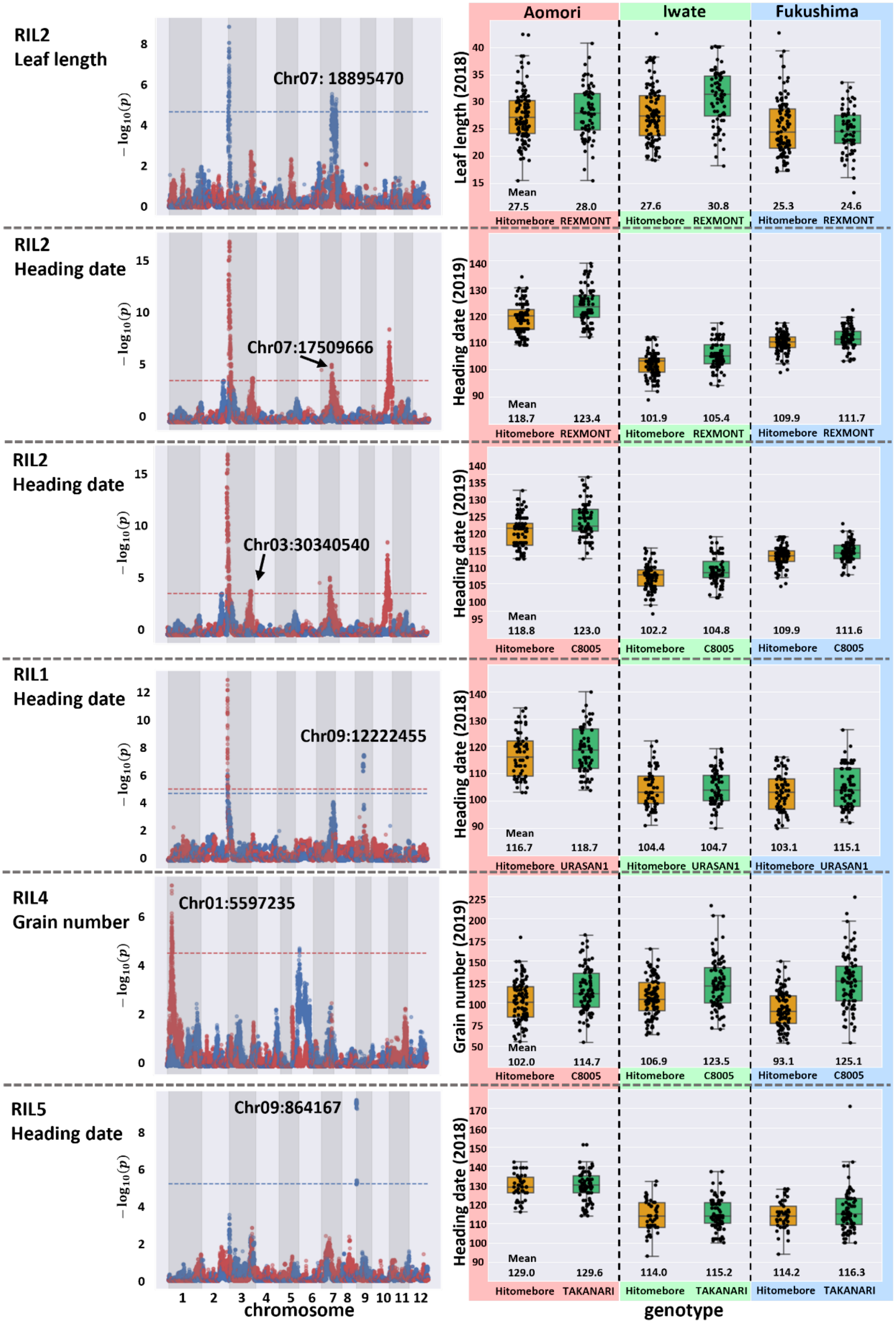

Figure S4 Identification of SNPs with GxE interaction effect in RIL1, 2, 3, 4, and 5 and the

variation of genetic effects in three locations. The left Manhattan plots shows the position of SNPs exhibiting GxE effect with statistical significance as revealed by the loglikelihood test comparing two alternative models with/without GxE interaction. The y-axis shows the –log10 (p) value of each SNP. The x-axis shows the genomic position. The colors show the trial years. The dashed lines indicate the significance, i.e., FDR <0.01. Boxplots in the right show the phenotypic values of RILs in three trial location with different SNP types at the genomic position identified in the left Manhattan plot. The horizontal line inside the box represents median value. Box range is the first and third quantile. The whisker extends to last datum less than the third quantile + 1.5*interquartile range (IQR) and the first datum greater than the first quantile—1.5*IQR. The X-axis shows the genotype of the SNP. The Y-axis shows phenotypic values. Each color frame indicates results from each trial location.
